# A clinical study of efficacy of polyglycolic acid patch in surgery for pneumothorax:a systematic review and meta-analysis

**DOI:** 10.1186/s13019-020-01137-8

**Published:** 2020-05-27

**Authors:** Yuang Mao, Zulei Zhang, Weibiao Zeng, Wenxiong Zhang, Jianyong Zhang, Guangmiao You, Yiping Wei

**Affiliations:** 1 Nanchang University, Nanchang, Jiangxi People’s Republic of China; 2 Department of Cardiothoracic Surgery, Second Affiliated Hospital of Nanchang University, 1 Minde Road, Nanchang, 330006 Jiangxi People’s Republic of China; 3 The Affiliated Hospital of Guizhou Medical University, Guiyang, Guizhou Province 550004 People’s Republic of China

**Keywords:** Polyglycolic acid, Pneumothorax

## Abstract

**Objectives:**

A polyglycolic acid (PGA) patch is often used in pulmonary bullae resection, but consensus has not been reached on its effect on patient recovery. The aim of the study is to conduct a systematic review and meta-analysis of studies of polyglycolic acid for bullectomy.

**Methods:**

A comprehensive literature search was performed using ScienceDirect, EMBASE, Ovid MEDLINE, PubMed, The Cochrane Library, Scopus, and Google Scholar. Clinical trials that compared PGA versus non-PGA for bullectomy were selected. The clinical endpoints included postoperative recurrence, average postoperative air leakage, prolonged air leaks, drainage tube removal time, and postoperative hospital stay.

**Results:**

A total of eight articles (1095 patients) were included. Compared to the non-PGA approach, the PGA approach was associated with lower rates of postoperative recurrence (95% confidence interval [CI]: 0.16 to 0.39, *p* < 0.00001),) and of prolonged air leaks (95% CI: 0.29 to 0.72, *p* = 0.0007); a shorter time of drainage tube removal (95% CI: − 1.36 to − 0.13, *p* = 0.02); The time of average postoperative air leakage, postoperative hospital stay and operative time did not show a significant difference between the two groups.

**Conclusions:**

These results suggest that the use of PGA patch might can prevent the postoperative recurrence of spontaneous pneumothorax and decrease the rates of prolonged air leaks. More large-scale, high-quality randomized controlled trials are required to confirm our finding.

## Introduction

Spontaneous pneumothorax (SP) is a common disease in thoracic surgery. Spontaneous pneumothorax can be divided into primary spontaneous pneumothorax (PSP) and secondary spontaneous pneumothorax (SSP) according to the presence of underlying lung diseases. PSP occurs predominantly in young and middle-aged men of lean height, and SSP occurs predominantly in elderly patients with underlying lung disease. Spontaneous pneumothorax (SP) is a common and relatively minor complication of thoracic surgery, usually requires surgical intervention; however, the troublesome problems are the high rate of recurrent pneumothorax and continual air leakage after the operation [1]. Video-assisted thoracic surgery (VATS) has gradually became a standard treatment choice for PSP since 1990s; in contrast to thoracotomy, it is beneficial in reducing scarring and pain and improving cosmetic outcomes, but VATS is flawed by its relatively high postoperative recurrence rate and prolonged postoperative air leakage [2–4]. Therefore, researchers have tried to develop an effective post-thoracoscopic bullectomy procedure. A bioabsorbable polyglycolic acid (PGA) patch has recently come into wide use in every surgical field [2, 5]. Some studies have concluded that staple line coverage with a PGA patch along the resection line after thoracoscopic bullectomy can inhibit postoperative recurrence and prolonged postoperative air leakage [6, 7]. While other studies shown that PGA patch was no significant difference in the number of air leakages lasting more than 3 days [8]. To determine whether staple line coverage with a PGA patch can decrease the rate of postoperative recurrence, we performed a systemic review and meta-analysis.

## Materials and methods

### Search strategy

The study was conducted according to the Preferred Reporting Items for Systematic Reviews and Meta-Analyses criteria (PRISMA) as shown in S1 File. On June 5, 2019, all relevant scientific literature published from January 2000 to June 2019 was identified by two investigators performing an extensive literature search. The following databases were searched: ScienceDirect, EMBASE, Ovid MEDLINE, PubMed, The Cochrane Library, Scopus, and Google Scholar. The MeSH terms “polyglycolic acid” and “pneumothorax or aerothorax” were used. The following inclusion criteria were used: (1) published in English, (2) clinical trials comparing PGA and non-PGA for pneumothorax, and (3) the most recent article or the article with the most detail was chosen when duplication of data was reported in more than one article. Excluded were case reports, meta-analyses, reviews without original data, letters, animal studies, and studies with duplicated data.

### Data extraction

The eligible studies were extracted data by two investigators; a third investigator helped in resolving any disagreement. The following recorded data included study design, first author, publication year, number of patients per group, nation, duration of enrollment, postoperative recurrence, average postoperative air leakage, prolonged air leaks, drainage tube removal time, Operative time, and postoperative hospital stay.

### Quality assessment of included studies

We assessed the quality of the included studies using the Jadad scale for randomized controlled trials (RCTs) and the Newcastle–Ottawa Scale (NOS) for nonrandomized studies. There are three main items in the Jadad scale (5 points): masking, randomization, and accountability of all patients. High-quality studies scored ≥3 points. Three items were analyzed in the NOS (9 points): comparability, exposure, and selection. We regarded those scoring 6–7 points as medium-quality studies and those scoring 8–9 points as high-quality studies.

### Statistical analysis

Review Manager 5.3 (The Nordic Cochrane Centre, The Cochrane Collaboration, Copenhagen, Denmark) and STATA 12.0 (StataCorp LP, College Station, TX, USA) was used to perform the meta-analysis. Statistical significance can appear if a *P*-value <0.5. The differences between two groups in discontinuous variables were assessed using relative risk (RR) with 95% confidence interval (95% CI), while those in continuous variables using analysis of variance. The heterogeneity of studies was evaluated by I^2^ and Cochran Q. An I^2^ < 50% or a *P*-value for the Q test > 1 was regarded as indicating acceptable heterogeneity and a fixed-effects model was used; otherwise, a random-effects model was used. We visually inspected the funnel plots to assess potential publication biases. Subgroup analyses by patient age < 30 years or > 40 years were performed.

## Results

### Search results and quality assessment of included studies

**Fig. 1:**
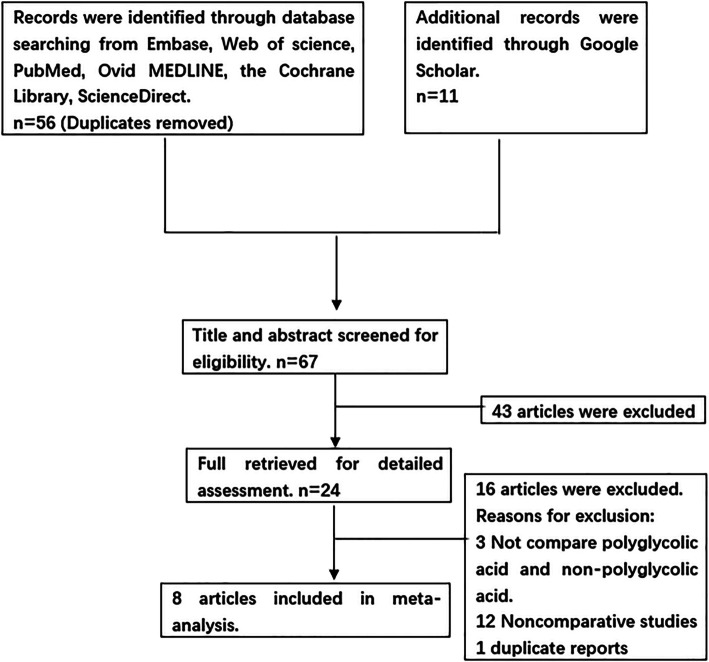
Flow diagram of study selection

From the reference list and database searches, we initially found 67 potentially eligible articles and selected eight studies with 1095 patients (599 in the PGA group, 496 in the non-PGA group) for final analysis (Fig. 1). Of these eight articles, five articles were retrospective studies, and the other three were RCTs. We used Jadad scales and NOS to assess scores: three articles were of medium quality, and five articles were of high quality. The main evaluation indices and the baseline characteristics of the included studies are shown in Table 1.

**Table 1:** Summary of the eight studies included in the present meta-analysis

Study	First	Country	Institution	Enrolled year		No. of patients		Outcomes	Design	Quality
	Author			PGA	Control	PGA	Control			
2003 [9]	Minami	Japan	Single	Not mentioned	Not mentioned	19	30	①	Retrospective	6
2011 [10]	Sun	China	Single	1996.06–2009.06	2009.06–2009.06	96	43	①④⑥	RCT	4
2012 [11]	Lin	China	Single	1995.01–2010.12	1995.1–2010.12	24	32	①④⑤	Retrospective	7
2013 [12]	Lee	Korea	Single	2009.01–2011.08	2009.01–2011.08	129	128	①②③④⑤	RCT	3
2015 [6]	Hirai	Japan	Single	1994.04–2007.12	1994.04–2007.12	181	98	①	Retrospective	6
2016 [2]	Hong	Korea	Single	2011.01–2013.04	2011.01–2013.04	58	58	①③④	Retrospective	8
2017 [8]	Zhang	China	Single	2015.01–2016.06	2015.01–2016.06	60	74	①②③④⑤⑥	RCT	4
2017 [13]	Kimura	Japan	Single	2008.03–2011.11	2008.03–2011.11	32	33	①③④⑥	Retrospective	6

① Postoperative recurrence, ② Average postoperative air leakage (days), ③ Prolonged air leaks (≧3–4 days), ④ Drainage tube removal time (days), ⑤ Postoperative hospital stay (days), ⑥ Operative time (minutes) RCT:randomized controlled trials

### Comparison of postoperative recurrence

**Fig. 2:**
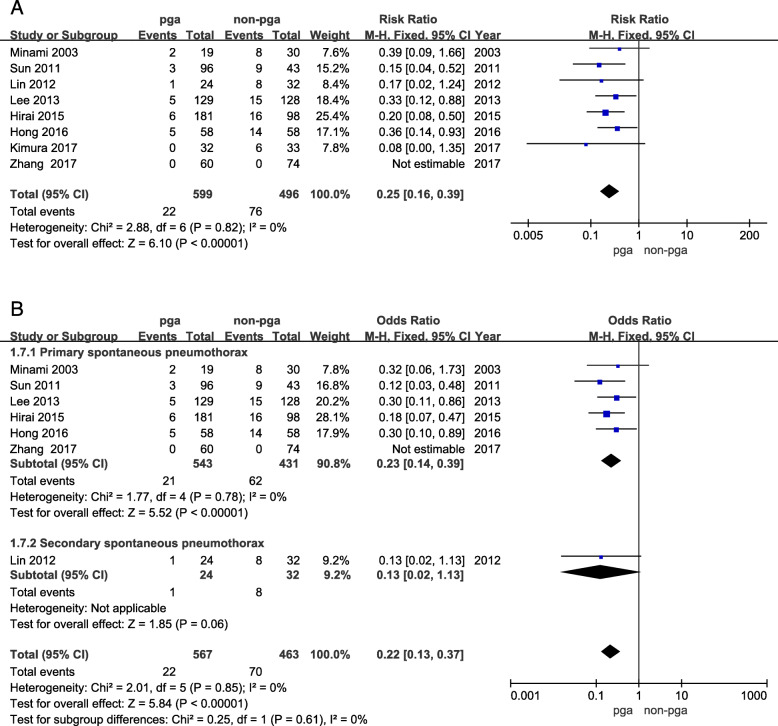
**a** Relative risks for PGA group and non-PGA group in comparison of postoperative recurrence. **b** Relative risks of subgroup for PGA group and non-PGA group in comparison of postoperative recurrence

All eight articles were evaluated for the rate of postoperative recurrence. There was no obvious heterogeneity between these studies (I^2^ = 0%, *p* = 0.82). The postoperative recurrence was 3.7% (22/599) and 15.3% (76/496) for the PGA group and non-PGA group, respectively. The pooled result showed that when compared to the non-PGA group, the PGA group showed a significantly lower postoperative recurrence (95% CI: 0.16 to 0.39, *p* < 0.00001), Fig. 2a). According to different types, seven articles (One article contains two types of spontaneous pneumothorax which are excluded) are divided into primary spontaneous pneumothorax and secondary spontaneous pneumothorax for subgroup analysis. The primary spontaneous pneumothorax subgroup contains six articles, and recurrence rate of PGA group was significantly lower than that of non-PGA group (95% CI: 0.14 to 0.39, *p* < 0.01), Fig. 2b). There was only one article in the secondary spontaneous pneumothorax subgroup, and the recurrence rate of PGA group was also significantly lower than that of non-PGA group (95% CI: 0.02 to 1.13, p < 0.01, Fig. 2b).

### Comparison of average postoperative air leakage(d)

**Fig. 3:**
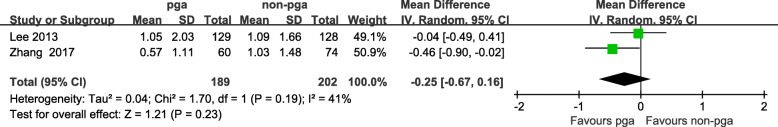
Mean difference for PGA group and non-PGA group in average postoperative air leakage

Two articles were identified for average postoperative air leakage comparison, which involved 189 patients in the PGA group and 202 patients in the non-PGA group. There was no obvious heterogeneity between these studies (I^2^ = 41%, *p* = 0.19). There was no significant difference between the PGA and non-PGA groups (95% CI: − 0.67 to 0.16, *p* = 0.23; Fig. 3).

### Comparison of prolonged air leaks

**Fig. 4:**
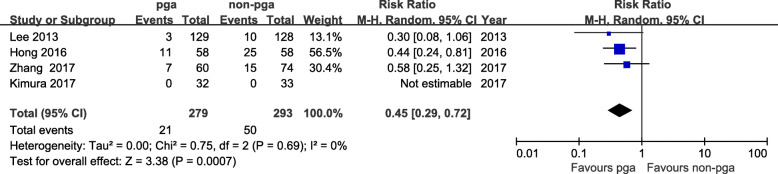
Relative risk for PGA group and non-PGA group in prolonged air leaks

Four articles were identified for comparison of prolonged air leaks, which involved 279 patients in the PGA group and 293 patients in the non-PGA group. There was no significant heterogeneity between these studies (I^2^ = 0%, *p* = 0.69). The incidence of prolonged air leaks was significantly lower in the PGA group than in the non-PGA group (95% CI: 0.29 to 0.72, *p* = 0.0007; Fig. 4).

### Comparison of drainage tube removal time

**Fig. 5:**
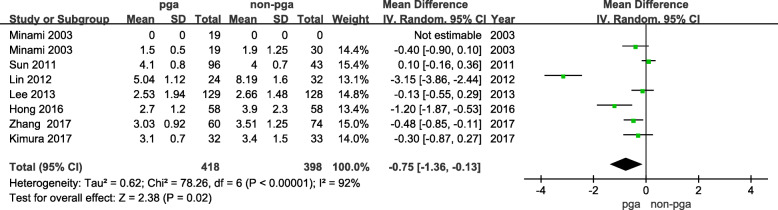
Mean difference for PGA group and non-PGA group in comparison of drainage tube removal time

Seven articles were identified for comparison of the drainage tube removal time; these involved 418 patients in the PGA group and 398 patients in the non-PGA group. There was significant heterogeneity between these studies (I^2^ = 92%, *p* < 0.00001). The drainage tube removal time was significantly shorter in the PGA group than in the non-PGA group (95% CI: − 1.36 to − 0.13, *p* = 0.02; Fig. 5).

### Comparison of postoperative hospital stay

**Fig. 6:**
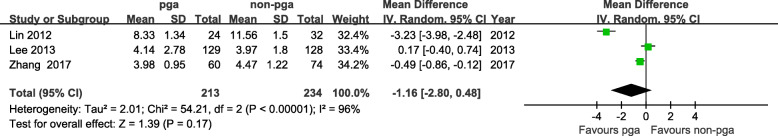
Mean difference for PGA group and non-PGA group in comparison of postoperative hospital stay

Three articles were identified for comparison of postoperative hospital stay; these involved 213 patients in the PGA group and 234 patients in the non-PGA group. There was significant heterogeneity between these studies (I^2^ = 96%, *p* < 0.00001). The pooled result showed there was no significant difference between the PGA and non-PGA groups (95% CI: − 2.8 to 0.48, *p* = 0.17,Fig. 6).

### Comparison of operative time (min)

**Fig. 7:**
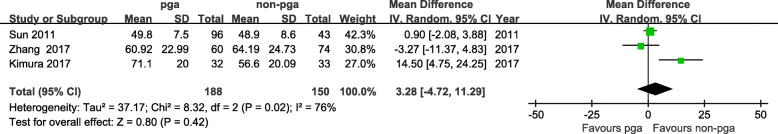
Mean difference for PGA group and non-PGA group in comparison of Operative time

Three articles were identified for comparison of operative time, which involved 188 patients in the PGA group and 150 patients in the non-PGA group. There was obvious heterogeneity between these studies (I^2^ = 76%, *p* = 0.02). There was no significant difference between the PGA and non-PGA groups (95% CI: − 4.72 to 11.29, *p* = 0.42; Fig. 7).

### Publication bias

**Fig. 8:**
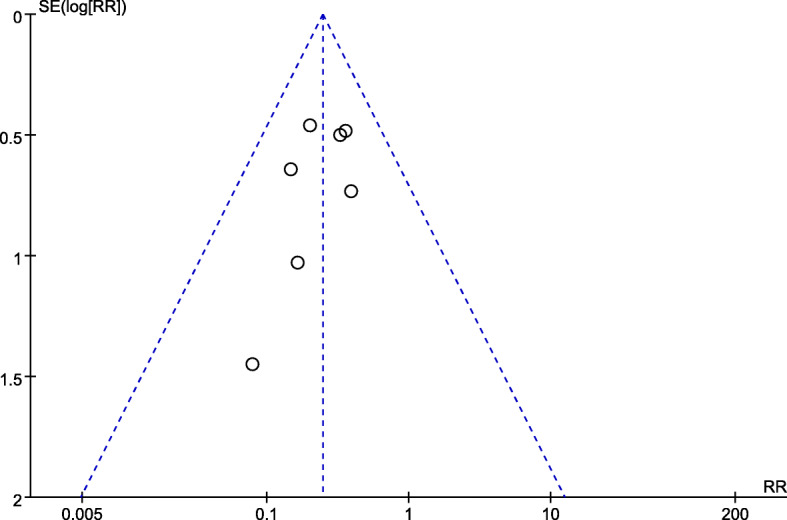
Funnel plot of relative risks for PGA group and non-PGA group in comparison of postoperative recurrence

The funnel plot with marked symmetry based on the data for postoperative recurrence suggests that there was no significant publication bias (Fig. 8).

## Discussion

Video-assisted thoracoscopic bullectomy has been a main treatment of spontaneous pneumothorax; however, according to the past several studies, the rate of recurrence is high, ranging from 9.4 to 24.5% [1, 3, 13, 14]. Although postoperative pneumothorax is not life-threatening, it can interfere greatly with patients’ quality of life, so it is important to decrease its rate of recurrence [4, 15, 16].

It is reported that various techniques have been used to decrease the incidence of postoperative recurrence, such as pleurectomy, apical pleural abrasion, chemical pleurodesis, and bioglue; however, pleurectomy may cause longer drainage tube removal time and intraoperative bleeding; in addition, apical pleural abrasion is associated with chest pain and risk of hemothorax or hematoma; a major disadvantage of chemical pleurodesis is tight pleural adhesions and severe postoperative chest pain; and bioglue may contribute to the transmission of infectious pathogens [2, 13, 17–19]. Bovine pericardium has also been reported to prevent air leakage; however, it can cause severe pleural adhesions, and it is difficult to preserve and prepare bovine pericardium [11].

PGA was introduced in the early 1970s as a new material for suture and has been combined with other biodegradable polymers for use in various fields of medicine [18, 20, 21]. The advantages of PGA are significant, namely, that it is nontoxic, biocompatible, and absorbable, and although it is reported that PGA can produce a mild inflammation, this reaction is rare; so far there are no relevant studies summarizing foreign body reaction to PGA [19, 22]. Despite these advantages, it has not been clear whether patients with pneumothorax can benefit from use of a PGA patch after surgery. Our study included 1095 patients with pneumothorax, which provide the most comprehensive evidence for postoperative recovery. Some scholars think that PGA can reduce the postoperative recurrence rate of pneumothorax. The first reason is that a PGA patch can decrease the rate of postoperative pulmonary fistula in the pleura. Second, a PGA patch can avoid new bullous formation and inhibit rupture of new bullae [13, 23, 24]. Our results showed that the incidence of postoperative recurrence is greater in the non-PGA group than in the PGA group; Considering the difference between primary spontaneous pneumothorax and secondary spontaneous pneumothorax, we conducted a subgroup analysis of two spontaneous pneumothoraxes, the results showed that PGA was equally effective in reducing the recurrence of pneumothorax.

Prolonged postoperative air leakage refers to patients with air leakage for more than 3–4 days after surgery. Prolonged postoperative air leakage is another postoperative complication, which can lead to pleural effusion, pulmonary atelectasis, pneumonia, or other complications. Some studies report that risk factors for postoperative air leaks are emphysematous changes in the stapler line or formation of new bullae; therefore, reinforcing the visceral pleura by covering the staple line would seem plausible as a way of preventing postoperative air leaks [3, 10, 14, 25]. However, in our comparison of average postoperative air leakage, there was no significant difference between the PGA group and the non-PGA group. On the other hand, we found that the incidence of prolonged air leaks was lower in the PGA group. Because the leakage time is not normal distribution, we guess covering the staple line with PGA is more beneficial to reduce postoperative air leaks of patients with poor lung. This result may suggest that covering the staple line with PGA can reduce postoperative air leaks.

In the comparison of drainage tube removal time, our results show that the time was shorter in the PGA group than in the non-PGA group. Lin et al. [11], who report on a clinical controlled study of 56 patients with pneumothorax due to silicosis, suggest that a long duration of postoperative chest drainage is related to persistent air leakage and tissue tearing by the sutures because of excessive tissue tension after thoracoscopic bullectomy. However, because the time of thoracic drainage tube in PGA group was shorter than in the control group (5.04 ± 1.12 vs. 8.19 ± 1.6, *p* = 0.00), we conjecture that PGA can reduce the time of pleural drainage by reducing postoperative air leaks. A long duration of postoperative chest drainage may lead to pulmonary infection, chest pain, and other complications. Zhang et al. [8] report that in a controlled clinical study of 134 patients with pneumothorax, there were only two complications in the PGA group, one lung infection and one atelectasis, while there were 12 complications in the control group. The time of using the thoracic drainage tube was less in the PGA group than in the control group, although the article shows PGA can reduce the complications, we conjecture that removing the thoracic drainage tube sooner reduces complications. There are studies that show that earlier removal of the drainage tube can result in a shorter postoperative hospital stay; However, this conclusion is the different as ours [26]. In comparison of postoperative hospital stay, we found there was no significant difference between the PGA group and the non-PGA group. We speculate that this result is due to different evidences of discharge from each doctor.

Our results shown that the chest tube time was significantly different between both groups while the postop air leak and length of stay were not. We believed the main reason was that the articles included for calculated postop air leak and length of stay were insufficient; Second, each doctor’s standard was different for judging whether there was air leakage, and statistics of air leaks was difficult. Moreover, the different criteria for discharged may also be one of the reasons. Therefore, PGA can reduce chest tube time, but its impact on the postop air leak and length of stay need to be verified by larger sample research.

A long operative time will affect the recovery of patients. In theory, preparation of PGA patches and intraoperative application could be expected to increase the operation time. However, in comparison of operative time, we found no significant differences between the PGA group and non-PGA groups. We believe operative time can be kept stable by having instrument nurses help prepare PGA patches.

Based on the analysis of the literature and data, we consider that the research has the following limitations. First, there are only 1095 people included in the literature, and most of them are retrospective research, among which only 3 RCTs affect the value of meta-analysis conclusions. Second, the main research subjects included in the literature are from Asian populations, mainly in China and Japan, which may result in some selection bias. Third, in calculating pneumothorax recurrence, the studies vary greatly in the follow-up time reported, which may indirectly lead to deviation in the final number, which may also affect the results. Finally, we were unable to compare important data such as intraoperative bleeding volume, and postoperative complications (pulmonary infection, heart rate arrhythmia, pleural effusion, etc.) because they are not reported in the relevant literature.

## Conclusion

In the treatment of pneumothorax by thoracoscopic bullectomy, the use of a PGA patch can reduce the recurrence of pneumothorax, and reduce the time of thoracic drainage and prolonged postoperative air leakage. Moreover, it does not increase operation time. But use a PGA patch can’t shorten the hospitalization time and average postoperative air leakage. Because of limitations of the included studies, however, this conclusion still needs to be verified by more high-quality RCT literature. Important data such as intraoperative bleeding volume and postoperative complications are expected to be discussed in more large-scale, high-quality RCTs in the future.

## Supplementary information

**Additional file 1: S1 File**. PRISMA checklist.

## Data Availability

Not applicable.

## Abbreviation

PGA: polyglycolic acid
PSP: Primary spontaneous pneumothorax
VATS: Video-assisted thoracic surgery
PRISMA: Preferred Reporting Items for Systematic Reviews and Meta-Analyses
RCTs: randomized controlled trials
NOS: Newcastle–Ottawa Scale
RR: relative risk
CI: confidence interval
